# Timing of Surgery and Medical Optimization in Multisystem Trauma: A Systematic Review of Outcomes Based on Internal Medicine Co-management With Orthopedic, Neurosurgical, Vascular, Thoracic, and General Surgical Emergencies

**DOI:** 10.7759/cureus.87262

**Published:** 2025-07-04

**Authors:** Abdelrahman Sahnon Abaker Sahnon, Mawada Taha, Yousif Osman, Abdelrahman Ibrahim, Ahmed S Ibrahim, Ahmed Mahdi, Jarallah H. J. Alkhazendar, Aliaa H Alkhazendar, Ahmed Mohamed, Shafaq Mushtaq

**Affiliations:** 1 General Practice, Speedy Recovery Clinic, Jeddah, SAU; 2 General Surgery, National Ribat University, Khartoum, SDN; 3 General Surgery, National Ribat University Hospital, Khartoum, SDN; 4 Trauma and Orthopedics, University Hospitals of North Midlands NHS Trust, Stoke-on-Trent, GBR; 5 Orthopedics and Traumatology, Royal Care International Hospital, Khartoum, SDN; 6 General Surgery, Albad'a General Hospital, Al Bad', SAU; 7 General and Emergency Surgery, East and North Hertfordshire NHS Trust, Lister Hospital, Stevenage, GBR; 8 Surgery, Islamic University of Gaza, Gaza, PSE; 9 Orthopedics and Trauma, Gezira Centre for Orthopaedic Surgery and Traumatology, Wad Madani, SDN; 10 Surgery, Liaquat National Hospital, Karachi, PAK

**Keywords:** advanced practice clinicians, co-management, hospitalist, internal medicine, multidisciplinary care, patient safety, perioperative care, surgical outcomes, surgical timing, trauma surgery

## Abstract

This systematic review explores the impact of internal medicine co-management on surgical outcomes and timing in adult patients across various surgical specialties, with an emphasis on trauma care. A comprehensive literature search identified five eligible studies comprising over 60,000 patients. Co-management models included hospitalist-led, advanced practice clinician-led, and multidisciplinary approaches. The outcomes consistently showed reductions in complications, hospital length of stay, rapid response team activations, and 30-day mortality. One study also highlighted indirect timing benefits, such as reduced intensive care unit (ICU) stay and ventilation days, with earlier surgical intervention. Although the evidence base is limited by study heterogeneity and lack of randomized trials, the consistent positive trends suggest that structured co-management enhances perioperative safety and system efficiency. These findings support broader implementation of co-management models, particularly in high-risk surgical and trauma populations, and underscore the need for further prospective research targeting surgical timing and interdisciplinary coordination.

## Introduction and background

Multisystem trauma is a complex medical condition. It involves injuries to multiple organs that often require urgent surgery [1]. These patients are usually unstable. Many have comorbidities, have low blood pressure, or need active resuscitation. Early surgery can help reduce intensive care unit (ICU) stay and time on a ventilator. But if the patient is not optimized, surgery can worsen inflammation and lead to organ failure. Therefore, the timing of surgery in trauma is very important [2]. It strongly affects patient outcomes in high-risk procedures like orthopedic fixation, craniotomy, thoracotomy, and vascular repair.

Internal medicine co-management has become more common in surgical care. It involves hospitalists, advanced practice clinicians (APCs), or full multidisciplinary teams (MDTs) [3]. These models help stabilize patients before surgery, reduce complications, and improve teamwork among care teams. They are especially helpful for high-risk or complex cases. Internal medicine teams can manage medical conditions before surgery and ensure the patient is ready [4]. However, there is limited data on how these teams affect the timing of surgery or readiness for the operating room. Most studies do not include patients from different surgical fields, like thoracic surgery, neurosurgery, vascular surgery, and trauma surgery, in one analysis.

This systematic review aims to evaluate the clinical outcomes associated with internal medicine co-management in multisystem trauma, with a focus on how co-management affects surgical timing and patient outcomes across diverse surgical specialties. By examining studies from orthopedic, neurosurgical, vascular, thoracic, and general surgical care, we seek to understand whether the integration of medical optimization through internal medicine teams facilitates earlier, safer surgical intervention and improves critical outcomes such as length of stay (LOS), complications, mortality, and resource utilization.

## Review

Materials and methods

Protocol and Reporting Standards

This systematic review was conducted in accordance with the Preferred Reporting Items for Systematic Reviews and Meta-Analyses (PRISMA) 2020 guidelines [5]. The review protocol was designed prior to initiation and followed a structured methodology to ensure transparency, reproducibility, and methodological rigor. The process included predefined eligibility criteria, a systematic search strategy, independent screening, and data extraction.

Eligibility Criteria and PICO Framework

Study selection was based on a clearly defined PICO framework [6]. The population (P) included adult surgical patients, primarily from orthopedic, neurosurgical, thoracic, or general surgical services, with special attention to trauma populations. The intervention (I) was internal medicine co-management, delivered via hospitalist physicians, APCs, or MDTs. The comparator (C) was standard surgical care without structured medical co-management. Outcomes (O) of interest included hospital LOS, mortality, ICU transfer, readmission rates, complication rates, and cost. Eligible studies included randomized controlled trials (RCTs), observational cohorts, pre-post intervention studies, and systematic reviews published in peer-reviewed journals in English. Studies focused exclusively on the timing of surgery were also included if relevant to the co-management context.

Search Strategy and Data Sources

A comprehensive literature search was conducted using PubMed, Embase, Scopus, and the Cochrane Library, covering articles published from database inception through April 2025. Keywords and Medical Subject Headings (MeSH) terms included combinations of "hospitalist", "co-management", "perioperative care", "internal medicine", "surgical outcomes", and "trauma surgery". Boolean operators were applied to refine the search, and filters for English language and human studies were used. Reference lists of included articles were hand-searched to identify additional eligible studies.

Study Selection and Screening

Two reviewers independently screened titles and abstracts, followed by a full-text review of potentially eligible studies. Discrepancies were resolved through discussion or the involvement of a third reviewer. A PRISMA flow diagram was constructed to document the study selection process, including the number of records identified, screened, excluded, and ultimately included in the review.

Data Extraction and Synthesis

Data were extracted independently by two reviewers using a standardized form, including author name, year of publication, study design, surgical specialty, population characteristics, co-management model, outcomes measured, and key findings. Studies were grouped by primary focus including co-management, timing, or both. Narrative synthesis was used to summarize the findings due to heterogeneity in study designs, populations, and outcomes.

Risk of Bias Assessment

Risk of bias was assessed using appropriate tools for each study type. Observational studies, including retrospective cohort and pre-post intervention designs, were evaluated using the ROBINS-I tool [7]. The included systematic review was assessed using the AMSTAR 2 tool [8]. All studies were determined to have either low or moderate risk of bias. Any discrepancies in the assessment were resolved by consensus.

Ethical Considerations

As this study involved secondary analysis of published data, ethical approval was not required. All included studies were from peer-reviewed sources, and no individual patient data were used.

Results

Study Selection Process

**Figure 1:**
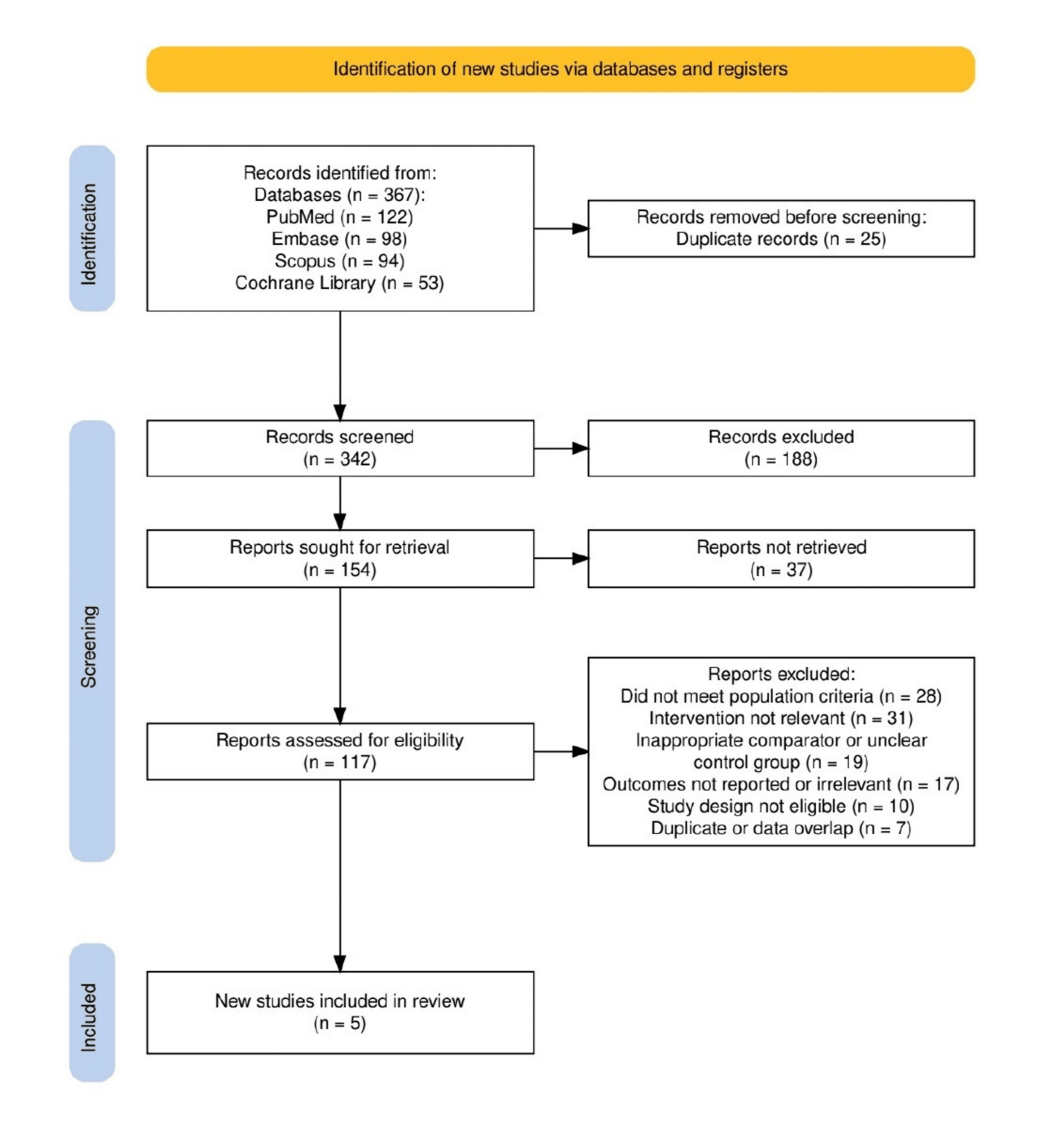
The study selection is illustrated through PRISMA flow diagram PRISMA: Preferred Reporting Items for Systematic Reviews and Meta-Analyses

A total of 367 records were identified through database searches, including PubMed (n=122), Embase (n=98), Scopus (n=94), and the Cochrane Library (n=53). After the removal of 25 duplicates, 342 records were screened by title and abstract, resulting in the exclusion of 188 studies. Of the 154 reports sought for retrieval, 37 could not be accessed in full. The remaining 117 full-text articles were assessed for eligibility, with 112 subsequently excluded for reasons including non-relevant population (n=28), intervention mismatch (n=31), inappropriate or unclear comparator (n=19), outcome irrelevance (n=17), ineligible study design (n=10), and data overlap or duplication (n=7). Ultimately, five studies met the inclusion criteria and were included in this systematic review. The complete study selection process is illustrated in Figure 1.

Characteristics of the Selected Studies

Table 1 summarizes the key characteristics of the five selected studies, covering a range of surgical specialties including orthopedic, neurosurgical, thoracic trauma, and mixed elective and emergency procedures. Study designs varied from retrospective cohort analyses and observational cohorts to systematic reviews. Populations primarily included adult surgical patients, with a focus on complex or trauma-related cases. Co-management models differed across studies, involving hospitalist physicians, APCs, and MDTs. Outcomes assessed included LOS, ICU transfers, complications, readmissions, mortality, and cost. Collectively, these studies highlight the impact of co-management on perioperative efficiency, patient safety, and system-level outcomes across diverse surgical settings.

**Table 1:** The characteristics of the selected studies in the systematic review LOS: length of stay; OR: odds ratio; MDT: multidisciplinary team; RCT: randomized controlled trial; ICU: intensive care unit; APC: advanced practice clinician; CMI: Case Mix Index; RRT: rapid response team; SSRF: surgical stabilization of rib fractures

Study (author, year)	Study design	Surgical specialty	Population description	Co-management model	Outcomes measured	Key findings
Shaw et al., 2020 [9]	Systematic review and meta-analysis (14 studies, including 1 RCT)	Mixed surgical specialties (elective and emergency procedures)	35,800 adult surgical patients across 14 studies (36.7% in intervention groups)	Internal medicine physician-only or MDT-based co-management	LOS, mortality, 30-day readmissions, complications, cost, functional outcomes	No overall difference in LOS or mortality; MDT co-management ↓ LOS (MD −2.03 days; p=0.05), ↓ mortality (OR 0.67; p=0.004); no effect on readmissions; complications/costs inconsistently reported
Johnson et al., 2024 [10]	Retrospective cohort study	Orthopedic joint and spine surgery	Adults (≥18 years) undergoing orthopedic joint/spine procedures (2014-2022)	Internal medicine APC-led co-management vs. usual orthopedic care	LOS, ICU transfer, return to OR, in-hospital and 30-day mortality, readmission, direct cost	APC co-management ↓ LOS (5%; p=0.009), ↓ return to OR (OR 0.51; p=0.002), ↓ 30-day mortality (OR 0.32; p=0.037); ↑ ICU transfers (OR 1.54; p=0.009); no difference in readmissions
Shi et al., 2025 [11]	Observational cohort, pre-post implementation	Orthopedic trauma surgery	Inpatients with orthopedic trauma procedures at a level 1 trauma center (2020-2024)	Orthopedic-hospitalist co-management vs. traditional consultation	CMI, LOS index, interdepartmental collaboration, process outcomes	Co-managed patients had ↑ CMI (2.58 vs. 2.29; p<0.0005); ↓ LOS index (0.92 vs. 1.06), not statistically significant (p=0.14); improved interdisciplinary collaboration and care processes
Rohatgi et al., 2020 [12]	Retrospective longitudinal analysis (2012-2018)	Orthopedic and neurosurgery	26,380 surgical discharges	Year-round dedicated hospitalist-surgeon co-management	Medical complications, LOS, RRT calls, cost	Over 6 years, ↓ odds of ≥1 medical complication (3.8%/year; p=0.01), ↓ LOS (0.3 days/year; p<0.0001), ↓ RRT calls (12.2%/year; p=0.001), ↓ direct cost ($3,424/discharge)
Becker et al., 2022 [13]	Matched-pairs retrospective analysis	Thoracic trauma (rib fractures)	Adults with multiple rib fractures from the German Trauma Registry	Focused on timing of SSRF (early vs. late)	Hospital LOS, ICU LOS, ventilation days	Early SSRF associated with ↓ ICU stay and ventilation days vs. late SSRF; hospital LOS was similar; emphasized benefits of timely surgery in multisystem trauma

Quality Assessment

Table 2 presents the quality assessment of included studies. Most were assessed using the ROBINS-I tool [7], with one evaluated via AMSTAR 2 [8] due to its review design. Overall, the studies showed a moderate risk of bias, largely due to their observational nature. However, appropriate adjustments, consistent findings, and transparent reporting support the reliability of their results.

**Table 2:** The risk of bias assessment of each of the selected studies using the AMSTAR 2 tool and ROBINS-I tool LOS: length of stay; OR: odds ratio; MDT: multidisciplinary team; RCT: randomized controlled trial; ICU: intensive care unit; APC: advanced practice clinician; CMI: Case Mix Index; RRT: rapid response team; SSRF: surgical stabilization of rib fractures; ROBINS-I: Risk Of Bias In Non-randomized Studies - of Interventions; AMSTAR 2: A Measurement Tool to Assess Systematic Reviews, version 2

Study (author, year)	Study design	Risk of bias tool used	Overall risk of bias	Justification
Shaw et al., 2020 [9]	Systematic review and meta-analysis	AMSTAR 2	Moderate	Transparent methodology, well-reported outcomes; moderate bias in included studies noted and discussed
Johnson et al., 2024 [10]	Retrospective cohort	ROBINS-I	Moderate	Propensity score weighting used; observational design limits residual confounding
Shi et al., 2025 [11]	Observational pre-post cohort	ROBINS-I	Low to moderate	Real-world data with limited confounding adjustment; findings consistent and plausible
Rohatgi et al., 2020 [12]	Longitudinal cohort	ROBINS-I	Low to moderate	Long-term data and trend analysis; adjustments performed; clear effect size trends over time
Becker et al., 2022 [13]	Matched retrospective cohort	ROBINS-I	Moderate	Matched design reduces confounding; well-structured reporting of surgical outcomes

Discussion

Across the five included studies, internal medicine co-management consistently demonstrated beneficial effects on clinical outcomes, notably in reducing hospital LOS, complication rates, and mortality. Shaw et al. [9], the largest synthesis involving over 35,000 surgical patients, reported that MDT co-management significantly reduced LOS by 2.03 days (p=0.05) and decreased mortality (OR 0.67; p=0.004). Effects on overall LOS and 30-day readmissions were variable but trended favorably. Johnson et al. [10] supported these findings in orthopedic surgery. APC-led co-management reduced LOS by 5% (p=0.009), halved return-to-operating-room events (OR 0.51; p=0.002), and decreased 30-day mortality by 68% (OR 0.32; p=0.037). Rohatgi et al. [12] observed longitudinal improvements over six years, including a 3.8% annual decline in medical complications (p=0.01), a 0.3-day/year reduction in LOS (p<0.0001), and fewer rapid response team (RRT) activations.

Shi et al. [11], focusing on orthopedic trauma, noted higher patient complexity under hospitalist co-management (Case Mix Index (CMI) 2.58 vs. 2.29; p<0.0005) without compromising LOS efficiency. Becker et al. [13] was the only study directly assessing surgical timing. Early surgical stabilization of rib fractures (SSRF) reduced ICU stay and ventilation days, though hospital LOS remained unchanged. Table 3 summarizes the comparative impact of internal medicine co-management on key outcomes across all included studies.

**Table 3:** Summary of key outcomes across included studies related to co-management APC: advanced practice clinician; LOS: length of stay; MDT: multidisciplinary team; OR: odds ratio; CMI: Case Mix Index; RRT: rapid response team

Study	Surgical specialty	Co-management model	Impact on LOS	Impact on mortality	Impact on complications
Shaw et al., 2020 [9]	Mixed (elective + emergency)	MDT and physician-only	↓ LOS by 2.03 days (MDT only)	↓ mortality (OR 0.67)	Mixed results
Johnson et al., 2024 [10]	Orthopedic	APC-led	↓ LOS (5%)	↓ 30-day mortality (68%)	↓ return to OR (OR 0.51)
Shi et al., 2025 [11]	Orthopedic trauma	Hospitalist-led	Favorable despite ↑ CMI	Not reported	↑ collaboration, process gains
Rohatgi et al., 2020 [12]	Orthopedic + neurosurgery	Year-round hospitalist co-management	↓ LOS (0.3 days/year)	↓ Medical complications (3.8%/year)	↓ RRT calls, ↓ costs
Becker et al., 2022 [13]	Thoracic trauma	Not defined; focused on timing	No change	Not reported	Not reported

Hospitalist- and APC-led co-management teams play a pivotal role in optimizing perioperative care [14]. By proactively managing chronic comorbidities, facilitating interprofessional communication, and expediting preoperative stabilization, these teams reduce complications and improve surgical readiness, especially in elderly or high-risk patients. The reductions in complication rates and 30-day mortality reported by Johnson et al. [10] and Rohatgi et al. [12] underscore the value of structured co-management in both elective and emergency settings. Becker et al. [13] also demonstrated the critical role of timely surgery in reducing ICU utilization, further supporting the need for early intervention frameworks guided by medical co-managers.

Moreover, co-management enhances hospital workflows and resource efficiency. In Shi et al. [11], co-management models improved early pain control, medication administration (e.g., bisphosphonates), and interdepartmental coordination. Rohatgi et al. [12] reported not only clinical benefits but also financial gains, including over $3,400 in savings per patient discharge. These system-level efficiencies reinforce the utility of co-management programs, particularly in high-acuity trauma and surgical cases where interdisciplinary coordination is vital [15,16].

Despite these strengths, several limitations must be acknowledged. The definition of co-management varied across studies, and outcome reporting lacked uniformity. The absence of RCTs and the predominance of observational data introduce potential confounding. Importantly, few studies clearly measured the timing of surgery as a discrete outcome, making it difficult to directly quantify co-management's effect on operative delays. Nonetheless, the consistency of improved patient-centered outcomes, shorter LOS, fewer complications, and enhanced coordination across diverse settings lends credibility to the observed benefits.

Future research should aim to address these limitations by employing standardized definitions of co-management, harmonized outcome measures, and high-quality study designs. A particular emphasis should be placed on measuring surgical timing metrics, including time to operating room, duration of preoperative optimization, and delay-related morbidity. Evaluating the relationship between co-management and time-sensitive surgical readiness may help identify actionable targets for quality improvement [17].

This review represents one of the first efforts to examine the relationship between internal medicine co-management and surgical timing across a broad spectrum of surgical specialties, including trauma. By synthesizing evidence from over 60,000 patients, it highlights the potential of co-management to improve not only individual outcomes but also system-wide efficiency. These findings support the broader integration of structured co-management into routine surgical care, particularly in high-risk and trauma populations, and lay the groundwork for more time-sensitive, interdisciplinary approaches in the perioperative landscape.

## Conclusions

This systematic review highlights the clinical and operational benefits of internal medicine co-management in multisystem surgical and trauma care. The key takeaway is that structured co-management, whether through hospitalists, multidisciplinary teams, or APC-led models, improves patient outcomes, reduces complications, shortens hospital stays, and enhances interdisciplinary coordination. Although surgical timing was not consistently reported, evidence suggests that co-management may indirectly reduce delays by optimizing patients earlier. These findings are relevant across multiple surgical specialties and support the integration of co-management models into routine practice, particularly in high-acuity settings. By synthesizing current data, this review fills a critical gap in the literature and emphasizes the need for standardized, time-sensitive approaches in perioperative care.
